# Supplementary material for: Chromothripsis during telomere crisis is independent of NHEJ, and consistent with a replicative origin
Source: Genome Res. 2019 May;29(5):737–49. doi: 10.1101/gr.240705.118 (PMC6499312; doi:10.1101/gr.240705.118)
Supplement: Supplemental Material [file supp_gr.240705.118_Supplemental_file_1.zip › contigs/annotated_contigs/DB93/contig.2.DB93_length_355_mean_cov_3.80281690141.docx]

**DB93_length_355_mean_cov_3.80281690141**

```
ATATATCGTCTCTATTAGTAAATAACAAATGATATACAAACCTCTGTAGAGAATCGTAAAACTTTAATAAGAGGCTTTAAAAAGACCAA
 >chr18:3123398-3123635 + E=2e-128
AACGAATGAAGAAAAATAACATGTTCATGGATTAGAAGGTTCAATATTTTTTAAATGTCAATTATTCCTTAATTTTTTTACAGATTCAA

TGCATCCTCAATCAGAATTCTAATGGGTTTAGTTGGGTTGAATGTAAGTTTTTTT|ATTG|CAAGTGGCAAGGGGAAGAGTCTTGGGAA
                                                        >chr3:7494942-7495064 - E=5e-62
CTAAGCCTCCAACTTGTGGGGTTTCACAGCATGCCAGATAGATCATGTAGGAATTGAAGTAGAGGACAGTCAGCTTGTGTCCACTGCAG

A
```
